# Achievements and Prospects in Electrochemical-Based Biosensing Platforms for Aflatoxin M_1_ Detection in Milk and Dairy Products

**DOI:** 10.3390/s17122951

**Published:** 2017-12-19

**Authors:** Ana-Maria Gurban, Petru Epure, Florin Oancea, Mihaela Doni

**Affiliations:** 1 Biotechnology Department, National Institute for Research & Development in Chemistry and Petrochemistry—ICECHIM, 202 Spl. Independentei, Sector 6, 060021 Bucharest, Romania; florino@ping.ro; 2 EPI-SISTEM SRL, Bvd Brasovului 145, Sacele, 505600 Brasov, Romania; petru.epure@epi.ro

**Keywords:** aflatoxin M_1_, immunoassays, antibody, aptamer, electrochemical biosensors

## Abstract

Aflatoxins, which are mainly produced by *Aspergillus flavus* and *parasiticus* growing on plants and products stored under inappropriate conditions, represent the most studied group of mycotoxins. Contamination of human and animal milk with aflatoxin M_1_, the hydroxylated metabolite of aflatoxin B_1_, is an important health risk factor due to its carcinogenicity and mutagenicity. Due to the low concentration of this aflatoxin in milk and milk products, the analytical methods used for its quantification have to be highly sensitive, specific and simple. This paper presents an overview of the analytical methods, especially of the electrochemical immunosensors and aptasensors, used for determination of aflatoxin M_1_.

## 1. Introduction

### Occurrence and Toxicity

Mycotoxins represent natural substances which occur as secondary product of the development of parasitic fungi, such as *Aspergillus*, *Fusarium*, *Penicillium*, *Claviceps* and *Alternania* genus in plants and products stored and then used for human and animal nutrition [1,2]. Usually, *Aspergillus* and *Penicillium* species grow on food and feeds during drying and storage conditions. Infections of crops with *Aspergillus* species can also occur in the field, during growing season, while *Fusarium* species represent destructive plant pathogens, producing mycotoxins in growing crops (corn, wheat and barley), before or post-harvesting [1].

Most of the mycotoxins are cytotoxic, producing a breakdown of cell membranes and preventing or influencing the DNA (deoxyribonucleic acid), RNA (ribonucleic acid) and protein synthesis, which poses significant risks to food safety [3]. Usually, they act by inhibition of protein synthesis at the ribosomes in cell, therefore cell division being inhibited too [4].

Contamination with these toxins is still considered unavoidable and unpredictable, even when good agricultural, storage and processing conditions are implemented. In order, to prevent the contamination of the crops, food and forage with aflatoxins, different strategies have been addressed. Chen et al. have reported the development of host resistance to aflatoxin producers, especially for maize crops [5].

Most of the time, human exposure to mycotoxins takes place through dermal contact, inhalation and ingestion. Mycotoxin ingestion may occur by eating contaminated food, direct via cereals or indirectly via animal products (e.g., eggs, milk and meat) [6]. Many mycotoxins present high stability against heating (baking, boiling), physical (sterilization, refrigeration, dehydration, desiccation, lyophilization and irradiation) and chemical treatments (extraction with solvents, modification of the molecular structure by oxidation, hydroxylation, etc.), they are largely resistant to industrial food processing [2,7]. Due to this resistance to processing, they can be found in all foodstuffs, their amount being just reduced by processing and not entirely eliminated.

Mycotoxins are relatively small molecules with total molecular weights of less than 500 Da, which can induce adverse health effects (carcinogenic, teratogenic, mutagenic, nephrotoxicity, hepatotoxicity and immunotoxicity) to humans and animals [8]. The impact of mycotoxins on the health of a given species depends on the amount and time of exposure, the age, weight, sex, diet and the presence of other mycotoxins [9,10]. Most of mycotoxins can cause four types of toxicity: chronic, carcinogenic, mutagenic and teratogenic. Most often, the described effects of the mycotoxins contamination are the affection of liver and kidney functions. Some of them have neurotoxic effects, being observed that their presence in small amounts may cause trembling in animals, while high amounts can cause brain damage or even death [7]. It has been demonstrated that long-term exposure to low doses of mycotoxins can induce cancer, especially of the liver and kidney [7,11,12]. Table 1 summarizes the effects observed over the humans and animals due to the mycotoxin contamination [11,12,13,14].

A number of factors such as caloric deprivation, vitamin deficiency, alcohol excess and infectious diseases status strongly influences the severity of mycotoxin contamination in humans [3].

Nowadays, there are more than 300 substances named mycotoxins, but it is not only difficult to define them, they are also difficult to classify. Usually, the classification criteria tend to follow the specialization of the person who is doing this. For example, chemists classify them by their chemical structure (e.g., coumarins, lactones, etc.), biochemists by their biosynthetic origins, clinicians by the affected organ (hepatotoxins, nephrotoxins, neurotoxins and immunotoxins), mycologists according to the fungi that produce them (e.g., *Aspergillus* toxins, *Penicillium* toxins, etc.) and not last, cell biologists classify them in four generic groups: carcinogens, teratogens, mutagens and allergens [3,7,8]. The International Agency for Research on Cancer (IARC) has defined four groups for classification of mycotoxins as carcinogenic or potentially carcinogenic to humans [15], as follows:

Group 1—carcinogenic to humans;
Group 2A—probably carcinogenic to humans (limited evidence on humans but sufficient in animals);
Group 2B—possibly carcinogenic to humans (limited evidence to humans and not sufficient evidence to animals);
Group 3—not classifiable as to its carcinogenicity to humans;
Group 4—probably not carcinogenic to humans.

Great attention has been paid to the aflatoxins, fumonisin, ochratoxin A, deoxynivalenol, patulin, zearalenone, trichothecenes and ergotamine, being considered that they are the most common contaminants found in food and animal feedstuffs, strongly affecting the human health and the economy [16,17,18,19]. It has been estimated that each year, about 25% of harvested crops worldwide are contaminated by mycotoxins, leading to significant economic losses [20].

A careful monitoring of these contaminants is required, thus, scientific advisory boards of the World Health Organization (WHO) with US Food and Drug Administration (FDA) and Food and Agriculture Organization (FAO) are responsible for the evaluation of mycotoxin-related contamination [3]. In the European Union (EU), the contamination of food with several mycotoxins is evaluated by European Food Safety Authority (EFSA) which has established maximum allowed limits for aflatoxins (AFs), ochratoxin A (OTA) and patulin (PAT) (EU regulation 466/2001) (European Commission, 2001) [21]. This regulation underwent several updates and was replaced in 2006 by EU regulation No. 1881/2006 [22], further updated in 2007 and 2010 by EU regulations No. 1126/2007 and No. 165/2010 [23,24].

Table 2 highlights the most important mycotoxins, the fungi that produce them, the group of their carcinogenicity, as well as the contaminated food and feedstuffs and the maximum admissible limits (MLs) for these compounds given by US-FDA and EU(EC2006) [25].

Contamination by mycotoxins is considerably influenced by several environmental factors, the geographic position and seasonal factors, such as temperature, humidity, pH and oxygen concentration, the same factors that affect the growth of toxic fungi. Thus, cultivation, harvesting, storage and the transport conditions of the crops and cereals are strongly related with level of mycotoxin contamination [3,9,19].

The chemical control of fungal growth and of mycotoxin biosynthesis in stored grains or other production stages can be achieved by using fumigation with oxidized ethylene, bromomethane [27] and 0.2 to 0.4% of ammonia [28]; insecticide treatments to prevent grain injury through facilitating its infection with toxigenic fungi and fungicidal treatments using some “natural” fungicides (e.g., chitosan). Good control for the mycotoxigenic fungi has been achieved under laboratory conditions using plant product (e.g., essential oils and extracts) as environmentally-friendly fungicides [29,30,31]. The use of some bacteria like *Bacillus* sp., propionic acid bacteria and lactic acid bacteria, seems to be a new opportunity for biological control of fungal growth and production of mycotoxins [32].

Great concern has been raised by the use of aflatoxins, satratoxins, trichothecene and fusarium toxins as biological warfare agents, since they are cheap and easy to access [33,34]. Aflatoxins have been used as biological weapon by Iraq in so-called “cancer bombs” [35]. Many countries considered that these mycotoxins have biothreat potential because of their effects over the nervous system function (the effects are temporary and/or reversible), inducing also the damage of the cell membranes (the effects are not reversible), therefore sensitive methods for their detection and monitoring being absolutely necessary [36].

## 2. Aflatoxins

Aflatoxins (AFs) represent the most toxic compounds from mycotoxins, having mutagenic and carcinogenic toxicity and contributing to human primary liver cancer, being classified as Group 1 carcinogens in humans [10,15]. They are secondary metabolites produced by *Aspergillus parasiticus* and *Aspergillus flavus*, which are present in soil and other organic materials. These fungi can grow on dried fruits (figs and raisins), on peanuts, ground nuts, corn, cottonseeds, coffee, cocoa, cereals (maize, wheat, barley, oats, rice), sunflower and soybeans seeds [37,38]. Sixteen aflatoxins have been identified, but only aflatoxins B_1_ (AFB_1_), B_2_ (AFB_2_), G_1_ (AFG_1_), G_2_ (AFG_2_) and M_1_ (AFM_1_) are currently analyzed [39]. *Aspergillus flavus* produces only aflatoxins B (AFB_1_ and AFB_2_), while *Aspergillus parasiticus* produces aflatoxins B (AFB_1_, AFB_2_) and aflatoxins G (AFG_1_, AFG_2_) [3,39].

Aflatoxin M1 represents the principal hydroxylated metabolite of AFB_1_, which can be detected in animal tissues and fluids (urine and milk). In the milk of mammals, aflatoxin M_1_ can be detected at 12 to 24 h after ingestion of contaminated food or feed with AFB_1_, its concentration which occur in excreted milk being correlated with the AFB_1_ levels found in the raw feedstuffs [40]. Contamination with AFM_1_ occurred also in dairy products from cow milk, especially in cheese, even at higher concentration than those found in raw milk. This is possible due to the stability of AFM_1_ towards the heating treatment involved in milk processing, binding well to casein [25,40].

**Figure 1:**
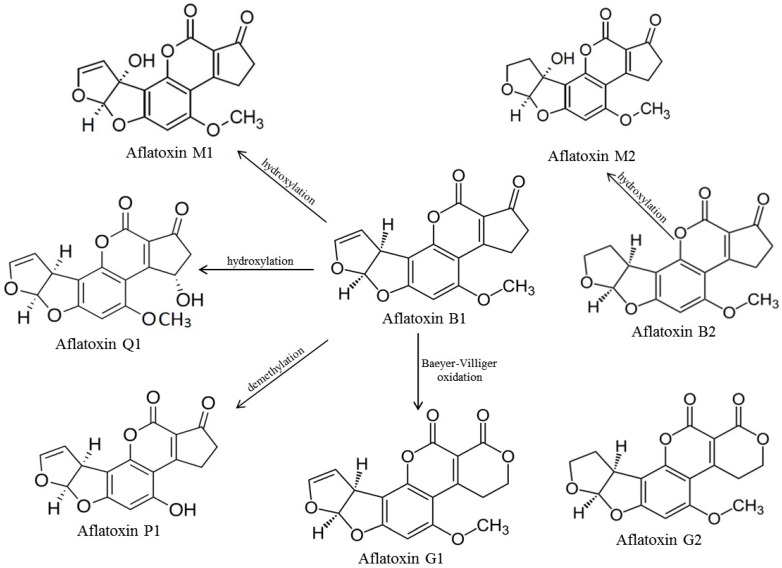
Chemical structures of aflatoxins and their metabolites.

The aflatoxins were named B and G based on their fluorescence colors under ultraviolet light (UV, 365 nm), AFB_1_ and AFB_2_ produce blue color, whereas AFG_1_ and AFG_2_ green color. They are difurano-coumarins derivatives, with low molecular weight which are soluble in solvents, such as methanol, chloroform and acetonitrile, having a wide spectrum of toxicity. Their chemical structures are drawn in Figure 1.

Aflatoxins are unstable to UV light, but highly stable to thermal treatments (e.g., baking, pasteurization, roasting) [41]. These mycotoxins can induce some types of cancer, hypoglycemia and elevated serum transaminase levels, being considered that aflatoxins are lipophilic molecules which are transported by blood stream and deposited in the hepatocytes [42].

**Figure 2:**
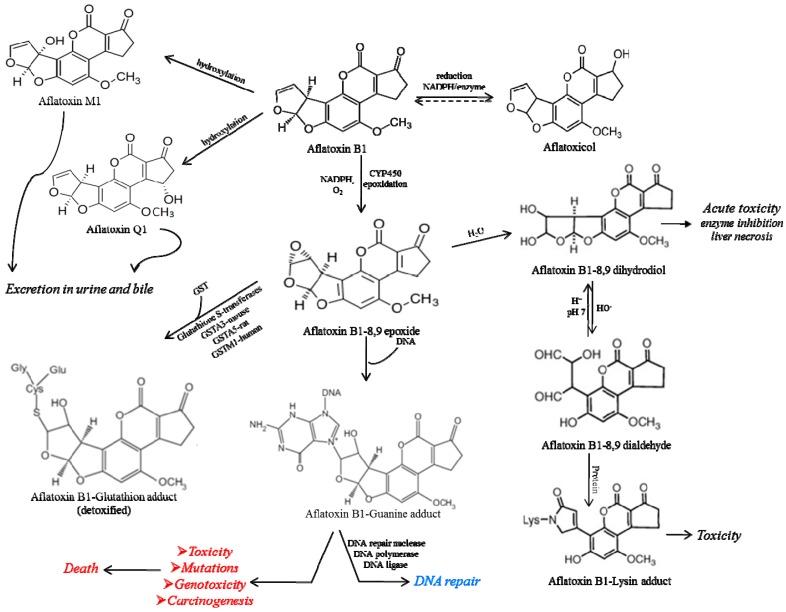
The biodegradation of aflatoxin B_1_ through metabolic pathways. DNA = deoxyribonucleic acid. NADPH = nicotinamide adenine dinucleotide phosphate. CYP450 = cytochrome P450. GST = glutathione-S-transferase.

After ingestion, the biodegradation of AFB_1_ takes place in liver, in an enzymatic system involving the hepatic microsomal cytochrome P450. The hydroxylated-AFB_1_ metabolite resulting from oxidation and demethylation of AFB_1_ is considered to be highly toxic, with mutagenic and carcinogenic effects [3,41,42]. A schematic representation of the aflatoxin B_1_ metabolism and the adduct formation are shown in Figure 2.

The epoxide form of aflatoxin B_1_ (AFBO), usually binds to proteins, to DNA and RNA, at the guanine base position in liver cells, modifying the genetic code that ensure the cell growth in the most active tissues (liver, intestine and bone marrow), and therefore leading to the appearance of genetic mutations and further to cancer [18,43].

The reduction of aflatoxin B_1_ by cytosolic reductase leads to formation of the aflatoxicol, a detoxification product which can be re-oxidized back to aflatoxin B_1_ by a microsomal dehydrogenase, increasing in this way the half-life physiological of AFB_1_ [44].

Other naturally occurring aflatoxins and their hydroxylated metabolites are not involved in the epoxidation reactions, and thus are considered to be less mutagenic and carcinogenic. Some of these metabolites are excreted in the urine of the contaminated individuals, this being used as an indicator for the individual exposure to aflatoxin B_1_ [18].

Up to now, the aflatoxin B_1_, found in high concentration in contaminated food and animal feed, is considered to be the most toxic compound among the aflatoxins. The level and duration of exposure to AFB_1_ are determinant factors for the toxic effects of this mycotoxin. Its lethal dose (LD_50_) for most species is considered to range from 1 to 50 mg/kg, with a critical toxicity level of less than 1 mg/kg for some highly susceptible species, such as poultry, rainbow trout and rats [45].

In humans, consumption of aflatoxin-contaminated food has been linked to different diseases, such as liver cancer, encephalopathy, pulmonary interstitial fibrosis, and effects on the reproductive and immune systems [2]. The incidence of hepatocellular carcinoma is directly related to aflatoxin consumption in diet, however, a quantification of the lifetime individual exposure is very difficult to predict. Liver cancer represents the most common type of cancer in Thailand, China, Philippines and many African countries, the incidence of this disease variating from one country to another [3,46,47].

### Aflatoxin M_1_

Aflatoxin M_1_ (AFM_1_) represents the principal hydroxylated metabolite of AFB_1_, biotransformed in the liver and excreted in the milk produced by mammary glands of lactating humans and animals, feed with AFB_1_-contaminated food [45]. Due to the high stability of AFM_1_ towards milk processing technologies, such as pasteurization and ultra-high temperature heating (UHT), and to other dairy product processing methods, this mycotoxin can be found not only in milk, but also in dairy products, usually at higher concentration than that found in raw milk [48,49].

Since milk, containing proteins, vitamins, minerals and fatty acids, is the most common nutrient, especially for infants and children, the presence of AFM_1_ in milk and dairy products represents an important health risk factor. The occurrence of AFM_1_ in human breast milk, milk and dairy products is of real public health concern, especially for infants and young children. It is considered that infants are more exposed to AFM_1_ contamination by breast milk intake than that using infant formula [50]. Thus, a maximum level of AFM_1_ in infant milk formula was set at 0.025 µg/kg by European Commission Regulation (EU 165/2010) [24], but the presence of AFM_1_ in human breast milk is not yet limited.

It has been observed that AFM_1_ can be detected in milk of lactating mammals after 12 h, with a peak of the concentration to 24 h from ingestion of AFB_1_-contaminated food. During 72 h from stopping the intake of AFB_1_-contaminated food, it has been observed a decreasing of the AFM_1_ concentration to undetectable levels [51].

Due to its semi-polar characteristics, the AFM_1_ binds strongly to casein, a phosphoprotein found in milk. Inactivation of AFM_1_ is difficult to achieve, being observed that the milk processing induced dramatic effects on AFM_1_ concentration [52].

Even if AFM_1_ is considered to be less mutagenic and carcinogenic than AFB_1_, its cytotoxicity has been studied in vitro using human liver microsomes and human cell line expressing or not expressing human cytochrome P450 enzymes. These experiments demonstrated a high toxic potential of AFM_1_ in the absence of metabolic activation, compared to AFB_1_ [53]. Thus, taking into consideration the toxic effect of AFM_1_, through DNA damage inducing gene mutation, chromosomal anomalies and cell transformation [40], the International Agency for Research on Cancer has changed the classification of this aflatoxin in 2002 from Group 2B to Group 1 [15].

As a result of all this, different analytical techniques for the detection and quantification of aflatoxins have been developed and reported in the literature, such as chromatography, UV-absorption, spectrometry, fluorescence and immunochemical assays. The choice of the analytical method must take into account different aspects for detection of these mycotoxins, such as target molecule, complex matrix, chemical characteristics, time of analysis and that limits of detection or/and quantification must be below the specific regulatory limits.

Further, this review will highlight the strengths and weakness of the different analytical methods developed and reported over the last period for AFM_1_ detection and quantification in milk and dairy products as a need for food safety monitoring and control.

## 3. Detection of AFM_1_

As was specified previously, the aflatoxin M_1_ presents high thermal stability to milk processing by sterilization, pasteurization or freezing. Therefore, the ingestion of milk and dairy products, the essential components of human diets, represents the first route of contamination with AFM_1_. The occurrence of AFM_1_ in milk and milk products take place in the range of ng/g and an early detection of this metabolite could be a relevant indicator of the risk factor to human health. Thus, there is still a continuous need for more sensitive, feasible, fast and affordable analytical methods for its detection and quantification.

Usually, most of the analytical methods require several steps prior analysis, such as extraction of AFM_1_ from its source, purification by removing other interference substances and quantification. Aflatoxins are soluble in organic polar solvents, such as methanol, acetonitrile, chloroform and acetone in different proportions, but in the case of immunoassay techniques special attention should be paid to the solvent used for extraction. A methanol-water mixture is preferred instead of acetone and acetonitrile, due to its less negative effect on antibodies [54].

Following the extraction, the purification and concentration of aflatoxin before its determination is performed by a clean-up step. The most common clean-up procedures used in aflatoxin analysis are solid phase extraction (SPE) and immunoaffinity column chromatography (IAC). Immunoaffinity chromatography involves high specificity and reversibility of the antibody-antigen binding and provides high selectivity and efficiency in separation and purification of the target analyte from complex matrices [55].

After the extraction and clean-up treatment by immunoaffinity columns, aflatoxin M1 is usually quantified using reference methods, such as high-performance liquid chromatography (HPLC) with fluorometric detection [56], thin layer chromatography (TLC) [57], liquid chromatography in tandem with mass spectrometry (LC/MS) [58], direct fluorescence measurement [59] and enzyme-linked immunosorbent assays (ELISA) [56,60,61].

### 3.1. Conventional Methods for Aflatoxin M_1_ Detection

Generally, the most common methods used for monitoring and detection of aflatoxins are: chromatographic and immunochemical methods, such as thin layer chromatography (TLC) [62], high-performance liquid chromatography (HPLC) [63], as well as enzyme-linked immunosorbent assay (ELISA) [56], sequential injection immunoassay and radioimmunoassay [64]. Immunochemical methods are based on specific antibodies and can be used with a good sensitivity for rapid screening of aflatoxins. Chromatographic methods are used for confirmation of the results obtained by rapid tests of screening, as well as for sensitive detection of the aflatoxins.

#### 3.1.1. Chromatographic Methods

Chromatography encompasses the most widely used techniques for separating a large number of analytes. These techniques are based on the physical interaction between a mobile phase and a stationary phase. As mobile phase liquid, gas or supercritical fluids can be used, hence the name of the corresponding chromatographic methods. Thin layer chromatography, HPLC and liquid chromatography coupled with different detectors are the main methods employed for analysis of mycotoxins [65]. Coupling these chromatographic methods with fluorescence or mass spectrometry detection allows for the sensitive determination of aflatoxin M_1_ in milk, cheese or other dairy products. Table 3 summarizes reported techniques coupled with different detectors used for AFM_1_ quantification.

##### Thin Layer Chromatography (TLC)

TLC is a traditional method used for the separation and determination of aflatoxins, reported for the first time by de Iongh et al. [66]. Using TLC, it was possible to determine several types of mycotoxins (e.g., aflatoxins, ochratoxins, patulin, tremorgenic toxins, zearalenone, citrinin, sterigmatocystins, versicolorins, etc.) in one sample, aflatoxins being detected in a range as low as 1 to 20 ppb [67,68]. Usually, the quantification of AFM_1_ is performed by its UV fluorescence, using an excitation at 360 nm and emission at 435 nm [67]. Even if this method is simple, fast and sensitive, it requires skilled personal, extensive sample pretreatment and expensive equipment [67,69]. Therefore, thin layer chromatography is no longer useful for the detection of AFM_1_, since its performance only allows determination at contamination levels too high or around the current regulatory limits of 0.05 ppb AFM_1_ [70].

##### High-Performance Liquid Chromatography (HPLC)

HPLC has been in under continuous development since the 1960s, being the most reported technique used for assessment of the aflatoxin status in contaminated food. HPLC coupled with different detectors, such as fluorescence, UV-Vis absorption or mass spectrometry represents the standard method used for quantification of the aflatoxin M_1_ in milk (liquid or powdered) and milk products [71,72].

Reversed-phase C-18 HPLC columns with fluorescence detection are commonly used for AFM_1_ determination, the fluorometric excitation and emission wavelengths being 360 and 435 nm, respectively [72,73]. Depending on the complexity of the matrix and in order to enhance the sensitivity for fluorescence determination of aflatoxins, usually a chemical derivatization is performed [73]. Since AFM_1_ is a naturally fluorescent compound, with an unsaturated furan ring, it can be either pre-column or post-column derivatized. An increase of the AFM_1_ sensitivity has been obtained by Chiavaro et al., by adding cyclodextrin to the methanol-water mobile phase. The authors managed to lower the detection limit for AFM_1_ to 0.0005 µg·kg^−1^, compared to detection limits (0.005–0.025 µg·kg^−1^) achieved by using official methods with pre-column derivatization and trifluoracetic acid [74].

However, these stages of derivatization have several drawbacks, including the use of toxic solvents, are time consuming due to the solvent evaporation, have limited stability and require daily maintenance.

Thus, to overcome the disadvantages of derivatization processes, HPLC was coupled with mass spectrometry, resulting efficient systems as HPLC-MS or HPLC-MS/MS for AFM_1_ detection. These methods use small amounts of sample for generating structural information and exhibit lower detection limits [75]. HPLC chromatographic techniques coupled with mass spectrometry are specific and selective are able to identify molecules by means of fragmentation patterns of spectral mass, sometimes involving just a single liquid extraction without any clean-up step.

However, all chromatographic methods are time consuming, expensive, requiring several complex sample pre-treatments steps and specialized personnel. These methods are suitable only for laboratory applications and not for in situ determination of AFM_1_, because usually the milk industries and dairy farms require a real-time and cheap monitoring of AFM_1_ in their products.

##### Fluorescence Spectrophotometric Methods

Since aflatoxins are fluorescent, the absorption process is followed by the emission of light under different wavelengths. Fluorescence is an important characteristic in the analysis of some molecules that emit energy at a specific wavelength, and therefore has been used for the determination of aflatoxins in animal feed and food grains [101]. Aflatoxins can be quantified in a range from 5 to 5000 ppb using spectrofluorometric methods in a short time, but the detection limit is quite higher than 4 µg/kg, the maximum limit set by the European Committee for total content of aflatoxins.

#### 3.1.2. Immunochemical Methods

Since the 1970s, the development of immunochemical methods for determination of aflatoxins has appeared as a solution to all the limitations of chromatographic and spectrophotometric methods. These methods, based on the specificity of antibody-antigen binding, are simple, sensitive, fast, less laborious and do not require highly trained personnel. Different immunochemical techniques were developed based not only on the high affinity and specificity of the antibodies for antigens, but rather using the affinity and specificity between receptors and ligands, too [101]. Usually, the enzymes, fluorophores and radioisotopes are used as labels for amplification of the signal recognition. Quantification of the antibody-antigen or receptor-ligand complexes formation is performed in correlation with the change in the absorbance of photons of light energy spectrophotometrically [102].

The use of aflatoxin-specific antibodies to form complexes with corresponding antigens has been applied for determination of aflatoxins, the high affinity and specificity of the antibody-antigen interaction leading to a high sensitivity and selectivity of the related assay [97]. Depending on the type of production, the antibodies can be divided into polyclonal (pAb), monoclonal (mAb) and recombinant (rAb) antibodies, but the monoclonal antibodies are the most frequent used for aflatoxin assays [103].

The most common immunochemical methods applied for aflatoxin assay are radioimmunoassay (RIA), enzyme-linked immunosorbent assay (ELISA), immunoaffinity column assay (ICA) and immunosensors (piezo, optical and electrochemical sensors) [97,98,104].

ELISA represents the most common method used for rapid screening of aflatoxins in medical diagnostic laboratories and research institutions. There are numerous commercially available ELISA test kits based on competitive enzyme immunoassay using alkaline phosphatase and horseradish peroxidase as labels [69,101,103]. In the last period, many publications have reported the analysis of aflatoxin M_1_ in different samples of milk (breast, cow, goat, etc.) using ELISA kit tests for screening of AFM_1_, with confirmation by the official HPLC method [52,64,91,92,105,106].

The ELISA technique is cheap, simple and sensitive, being suitable for a large number of samples at the same time. However, it is still laborious, requiring multiple washing steps and long incubation time, thus, it is a time-consuming method.

**Figure 3:**
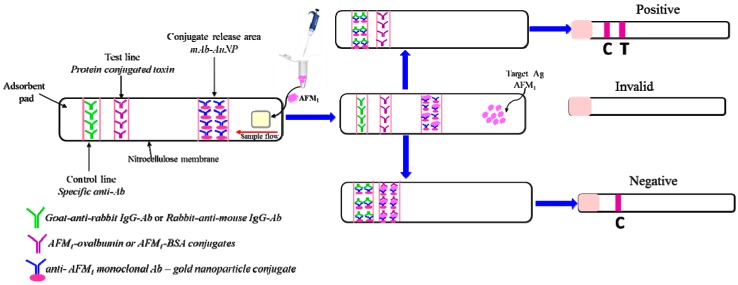
Detection of aflatoxin M_1_ (AFM_1_) using immunostrip.

Immunostrips or immunodipsticks have been developed for the rapid screening method of the low-molecular weight AFM_1_. The antibodies in this case are conjugated with gold nanoparticles which provide a visual red colored of binding zone [107,108,109,110,111]. The principle of the assay is based on the specific and sensitive interaction between liquid test sample containing aflatoxin and antibody-gold nanoparticle conjugates along membrane strips (Figure 3). The sample suspends the gold nanoparticles and aflatoxin binds to these particles coloring in red the binding-line.

This method is simple and efficient for on-site detection of AFM_1_ in milk samples, providing results in less than 10 min. The main disadvantage of these immunostrips is that each one can only be used for a single sample and large amounts of AFM_1_-conjugates are required for each assay, increasing in this way the assay cost [111].

### 3.2. Immunosensors

Immunosensors represent another direction that has grown in the last decades in development of sensitive, selective, simple and reliable systems for aflatoxins detection. Different biosensors based on optical (surface plasmon resonance, chemiluminescence, evanescent wave-based fiber optics) [112], surface acoustic wave (quartz crystal microbalance) [90], piezoelectric and electrochemical principles have been reported for AFM_1_ detection [112,113]. For the development of an immunosensor usually an antigen or an antibody coupled with a physical transducer such as gold, carbon or graphite is used as biological recognition element, which allows the detection of the binding species [101].

#### 3.2.1. Electrochemiluminescence

Electrochemiluminescent immunoassay (ECLIA) represents another highly sensitive and selective technique combining the analytical advantages of electrochemiluminescence, such as sensitivity, absence of the background optical signal and a facile control by changing the electrode potential with the specificity of the immunoassay. By using this technique, it is possible to detect ultratrace amounts of AFM_1_ in food. For the development of sensitive electrochemiluminescent immunoassay sensors, it is very important to define a signal tag labeled with the AFM_1_ antibody [104]. For this purpose, Ru(bpy)_3_^2+^ and luminol, as well as strongly luminescent semiconductor quantum dots (CdS, PbS, CdTe and ZnS) QDs have been extensively used for applications in biological imaging and labeling, having excellent optoelectronic properties [114,115]. It has been reported that the electrochemiluminescent signal was considerably amplified by using carbon nanomaterials, more specifically, carbon nanotubes (CNT). The surface of hybrid particles (QDs-CNT) can be labeled with attached antibodies as tags [93]. Thus, monoclonal antibodies of AFM_1_ have been immobilized on CdTe QDs-CNT composites for the development of ECLIA-based biosensors. Graphene oxide was chosen as absorbent material for AFM_1_ and further to conjugate with AFM_1_-monoclonal antibody/CdT QD-CNT, in order to form a sandwich immunocomplex GO/AFM_1_/AFM_1_-Ab/CdTeQD-CNT, which can generate an electrochemiluminescence signal on the electrode. Graphene oxide can be used also as absorbent material for other aromatic organic compounds, but the signal tag labeled with AFM_1_-Ab can react only with absorbed AFM_1_, thus the electrochemiluminescent signal will reflect only the amount of AFM_1_ absorbed onto graphene oxide [116].

Gan and co-authors have reported an electrochemiluminescent immunoassay for AFM_1_ detection in milk, by using magnetic Fe_3_O_4_-graphene oxide as absorbent for AFM_1_ and AFM_1_ antibody-labeled CdTe quantum dots as the signal tag based on a carbon screen-printed electrode. The authors reported a great enhancement of the electrochemiluminescent signal by using this immunocomplex, detection of AFM_1_ from milk samples being possible with a detection limit of 0.3 pg/mL [93].

#### 3.2.2. Electrochemical Immunosensors

In the development of electrochemical immunosensors devoted to AFM_1_ detection different types of bioreceptors on sensor platforms have been used, including antibodies, nucleic acids, protein ligands, prokaryotic and eukaryotic living cells and aptamers. Several electrochemical detection methods, such as amperometry, potentiometry and conductometry have been used in designing AFM_1_-based immunosensors, the most commonly used being amperometric methods. The good sensitivity, the reduced cost and the possibility of miniaturization of the developed amperometric immunosensors have been reported [117]. In designing electrochemical immunosensors, different electrodes materials were used as sensor platforms, the most commonly encountered materials being platinum, gold and various forms of carbon [118].

The possibilities of mass fabrication, low cost and single drop assays have attracted increasing interest in screen-printed technology. Also, the miniaturization of electrochemical sensor platforms leads to considerable lower sample consumption, and moreover, the combination of the electrochemical detection methods with the progress in sensor technologies makes these electrochemical immunosensors suitable for integration in point-of-care and portable devices as well as for in situ applications [119].

Using electrochemical immunosensors, the detection of aflatoxins can be performed in a direct competitive assay, in which a specific antibody is immobilized on the electrochemical transducer and the competition is carried out between the labelled and non-labelled analytes to bind with the specific antibody.

Thus, an amperometric disposable immunosensor have been designed Micheli et al. for AFM_1_ detection in raw milk, based on immobilization of a monoclonal antibody on a carbon screen-printed electrode (SPE) [97]. A direct competition between free AFM_1_ and it’s conjugate with peroxidase (AFM_1_-HRP) for the specific antibody was allowed to occur. 3,3′,5,5′-Tetramethyl- benzidine was used as enzymatic substrate for evaluation of the AFM_1_-HRP amount which reacted with the immobilized antibody, and electrochemical detection of the electroactive product was performed by chronoamperometric measurements at −0.1 V. A detection limit of 25 ppt for AFM_1_ was obtained using the disposable immunosensor, working in a range from 30 to 160 ppt. The authors proved that using electrochemical detection a better detection limit and shorter analysis time could be achieved [97].

Another immunosensor based on an antibody-modified carbon screen-printed electrode has been reported by Parker and Tothill, using a competitive ELISA assay constructed at the surface of a carbon paste electrode [109]. Using the competition between free AFM_1_ from samples and an AFM_1_-horseradish peroxidase conjugate for a monoclonal AFM_1_-antibody and electrochemical detection based on 3,3′,5,5′-tetramethylbenzidine (TMB)/hydrogen peroxide (H_2_O_2_) for HRP, the authors obtained a limit of AFM_1_ detection of 39 ng·L^−1^, with a dynamic range up to 1000 ng·L^−1^. The developed immunosensor was comparable in term of sensitivity with the common methods (ELISA, HPLC), but presented superior characteristics in terms of portability and cost [109].

An automated flow-injection immunoassay system has been developed for determination of AFM_1_ in raw milk samples by Badea et al. [98]. This system is characterized by an off-line incubation of a mixture containing the antigen (AFM_1_), fixed amounts of specific antibody to AFM_1_ (anti-AFM_1_) and peroxidase marked AFM_1_ (AFM_1_-HRP) until the equilibrium was reached, and then injected into the flow system. A column containing Protein G was used for separation of the free tracer and of the antibody-conjugate, while antibody-antigen complex was retained in the column due to the high affinity of the Protein G for the constant region of immunoglobulins. The activity of the enzymatic label horseradish peroxidase (HRP) has been evaluated by amperometric measurements using 3,3’,5,5’-tetramethylbenzidine. The flow-injection immunoassay system showed good reproducibility and short time of analysis, with low cost instrumentation, being easy to operate and the results being comparable with those obtained by HPLC [98].

A competitive immunoassay was used for development of an electrochemical sensor for detection of AFM_1_, based on magnetic nanoparticles (MNPs) coated with anti-AFM_1_ antibody [120]. The samples containing AFM_1_ were incubated with fixed amount of MNP-Ab and AFM_1_-HRP conjugate until equilibrium was reached, and afterwards the mixture was deposited onto the surface of screen-printed electrodes. The enzymatic response was amperometrically determined using 5-methylphenazinium methyl sulphate as mediator, the detection limit achieved using this immunosensor being 0.01 ppb. The system allowed determination of AFM_1_ directly in milk, after a simple centrifugation step, without any dilution or pretreatment steps, the analysis time being considerably reduced [120].

Dinckaya et al. have used a DNA biosensor for AFM_1_ detection in milk and dairy products, based on immobilization of a thiol-modified single strained DNA (ss-HSDNA) that bound specifically AFM_1_, using a self-assembled monolayer of cycotiamine and gold nanoparticles prepared onto a gold electrode [104]. The specific binding of AFM_1_ to ss-HSDNA has been studied by cyclic voltammetry and electrochemical impedance spectroscopy (EIS), and using this biosensor the detection of AFM_1_ was possible to be performed in a linear range of 1 to 14 ng·mL^−1^ [104].

Another impedimetric immunosensor for detection of AFM_1_ in milk has been developed by Bacher et al. [121]. This immunosensor is based on functionalization of a silver (Ag) wire electrode with selective monoclonal antibody of AFM_1_ using a self-assembled monolayer (SAM) of 11-marcaptoundecanoic acid (11-MUA). Electrochemical impedance spectroscopy was used for analyzing the electrical properties of the modified electrode, when an antibody coupled to the electrode reacts with its specific antigen. It was shown that the applied potential strongly influences the antibody-antigen interaction. The limit of detection obtained using this impedimetric immunosensor was 1 pg/mL, with short time of analysis of about 20 min, while the sensitivity was about 2.1% impedance change per decade. The authors reported a period of use of the bio-functionalized silver-wire sensor for up to two weeks [121].

Biosensors based on cells for detection of estrogenic toxins represent another important direction in analytical science evolution due to the high sensitivity, fast rate detection, low cost and the possibility of one target analyte detection [122,123]. Usually, for development of such biosensors, viable whole cells which are able to recognize a particular analyte or a group of analytes are recruited as sensing element. In this sense, bacteria, yeast or eukaryotic cells, including vertebrate or mammalian cells, can be used for development of cells-based biosensors [122]. The use of a genetically modified *Saccharomyces cerevisiae* strain for the detection of estrogenic mycotoxin residues in milk was reported by Valimaa et al. [123].

Larou et al. have developed a biosensor based on mammalian cells containing membranes engineered by artificial electro-insertion of AFM_1_-specific antibodies [124]. This biosensor provides an electric response of the membrane-engineered fibroblast cells suspended in an alginate gel matrix, due to the change of their membrane potential after the interaction between AFM_1_ and its specific antibodies. Thus, detection of AFM_1_ at concentrations as low as 5 ppt in just 3 min was possible, the assay being selective for AFB_1_ and OTA [124].

Recently, the development of aptamer-based biosensors for mycotoxin detection has received considerable attention, having several advantages such as low cost, high stability and sensitivity, and the fact they can be easily synthetized and modified compared to antibodies. Aptamers are functional short oligonucleotides, reported for the first time in 1990 [125,126], selected in vitro from combinatorial libraries, which can bind with high affinity and specificity to a wide range of target molecules (proteins, toxins, drugs, organic or inorganic molecules, etc.) [127]. The process of in vitro selection by which these oligonucleotide ligands are obtained is called Systematic Evolution of Ligands by Exponential enrichment (SELEX) [127,128]. The high specificity of the aptamers is a result of the selective step in the SELEX process called “counter-SELEX” that eliminates the sequences that also bind to closely-related analogs of the target [127]. Malhotra and co-authors reported for the first time a specific aptamer to AFM_1_ having a dissociation constant (Kd) of about 35 nmol·L^−1^ [129].

Detection of AFM_1_ can be performed with aptasensors using electrochemical and impedance spectroscopy detection [94,128,130]. An impedimetric aptasensor has been designed by Istamboulier and co-authors for determination of AFM_1_ in milk based on DNA-aptamer recognition element and electrochemical impedance spectroscopy detection [130]. The AFM_1_-aptamer (a 21-mer DNA oligonucleotide) was covalently immobilized on the surface of carbon screen-printed electrodes through carbodiimide immobilization procedure, after a previous activation of the electrode surface with diazonium salt. The interaction between aptamer and AFM_1_ induced an increase of the electron transfer resistance at the electrode surface, allowing in this way determination of AFM_1_ with a detection limit of 1.15 ng·L^−1^. A simple preliminary treatment of the milk samples was carried out, by filtration through a 0.2 µm polytetrafluoroethylene (PTFE) membrane of the mixture containing milk sample, methanol and binding buffer [130].

Another aptasensor for AFM_1_ detection was designed by Guo et al. using the interaction between a specific aptamer to AFM_1_ with biotin-streptavidin and its complementary ssDNA as template for a real-time quantitative polymerase chain reaction amplification [94]. This aptasensor has been used for determination of AFM_1_ in infant rice cereals and infant milk powder samples, showing a high selectivity to AFM_1_ over other aflatoxins, the detection limit obtained being 0.03 ng·L^−1^ [94].

Nguyen and co-authors attempted to improve the sensitivity of AFM_1_ detection by using covalent immobilization of specific aptamers on COOH- functionalized magnetic nanoparticles [128]. The magnetic nanoparticles incorporated in polyaniline film were polymerized on the surface of an interdigitated electrode as sensitive film for an AFM_1_-based electrochemical biosensor. Direct detection of AFM_1_ was performed at the Fe_3_O_4_/polyaniline interface by cyclic and square wave voltammetry, with good sensitivity and a detection limit of 1.98 ng·L^−1^. The developed aptasensor allowed the detection of AFM_1_ below the legislative set limits, with several advantages over other common analytical methods, such as sensitivity, stability, label free format, low analysis time and cost effectiveness [128].

#### 3.2.3. Optical Immunosensors

Optical Waveguide Light mode Spectroscopy based on amino functionalized integrated optical waveguide sensors was used for quantitative determination of AFM_1_ in milk samples [131]. The covalent immobilization of AFM_1_-HRP conjugate was carried out on the surface of the amino functionalized SiO_2_-TiO_2_ based sensor using glutaraldehyde. The specific antibody to AFM1 was added in the sample and further measured by the immobilized antigen. For regeneration of the sensor surface was used HCl 10 mM, acting for dissociation of the immunocomplexes. Milk samples were analyzed using three different types of pre-sampling preparation (filtration, centrifugation and size exclusion centrifugation), AFM_1_ being determined in a dynamic range from 0.001 to 0.1 ng·mL^−1^ [131].

Lou and co-authors have developed a wave-based optofluidic biosensing platform for sensitive detection of aflatoxin M_1_ in dairy products [132]. The portable, miniaturized device consisted in an optical fiber biosensor modified with AFM_1_-Ovalbumin (ovalbumin) embedded in a poly-methyl-methacrylate-based optofluidic cell and a pulse diode laser was used to excite the fluorescence-labelled antibody. The fluorescence signal was linearly dependent on AFM_1_ concentration allowing its direct quantification. Using the developed device, a detection limit of 5 ng·L^−1^ was achieved for AFM_1_ in dairy products [132].

Surface plasmon resonance (SPR) represents another optical technique used for immunoassay analysis of mycotoxins. The principle of detection using SPR platforms is based on measurements of changes in refractive index produced when the target analyte binds to its specific antibody immobilized on the sensor surface [101]. The SPR immunosensor has been used for detection and quantification of aflatoxin B_1_ or for multiple detection of mycotoxins [133]. The SPR immunosensors obtained by immobilization of monoclonal antibodies encountered serious problems concerning the regeneration of the sensor surface, due to the high affinity binding of the monoclonal antibodies [133].

## 4. Conclusions

By presenting different analytical methods for determination of aflatoxin M_1_ in milk (animal, human or powdered) and dairy products we have shown that electrochemical biosensing platforms offer highly sensitive and specific alternatives to the conventional methods.

While TLC, HPLC and enzyme-linked immunosorbent assay are considered the gold standard methods for AFM_1_ determination, they are still more cumbersome, expensive and time-consuming techniques. Thus, electrochemical immunosensors represent a suitable alternative for AFM_1_ detection, offering several advantages, such as versatility, high sensitivity, low production cost, easy modification and good stability. The use of screen-printed electrodes in combination with monoclonal antibodies or aptamers as bioreceptors leads to miniaturization of the system and to an improvement of the sensitivity, speed and low cost of analysis.

In addition, an oriented immobilization of biomolecules can be achieved due to their small size, simplicity and easy functionality, allowing in this way an increase of the binding efficiency and minimizing non-specific adsorptions on the biosensor surface.

Since aflatoxin B_1_ contamination of feed and food products still remains a matter of increasing concern, aflatoxin M_1_ contamination of milk and milk products will continue to be a risk factor for humans and especially for infants and young children. Taking into consideration the rapid and continuous development of the analytical methods and nanotechnology, different approaches for aflatoxins analysis will continue to be developed.

## Figures and Tables

**Table 1:** Symptoms and effects occurred in humans and animals by mycotoxins contamination.

System	Vascular	Digestive	Respiratory	Nervous	Skin	Reproductive and Excretory
Symptoms/Effects	Increased fragility of blood vessels	Vomiting Intestinal hemorrhage	Shortness of breath	Tremor/Lack of coordination	Irritation	Infertility
	Internal hemorrhage to mucous and lungs	Liver necrosis	Bleeding of lungs	Depression	Burning sensation	Nephrotoxicity
		Mucous membrane destruction		Headache	Photosensitivity	

**Table 2:** The most common mycotoxins, their toxicity and limitation levels in food and feedstuffs.

Mycotoxin	Fungal Source	Group of Toxicity [25]	Contaminated Food	US-FDA MLs [26] (µg/kg)	EU-EFSA MLs [24] (µg/kg)
Aflatoxins (B_1_, B_2_, G_1_, G_2_)	*Aspergillus flavus* *Aspergillus parasiticus*	1	Wheat, maize, rice, peanut, pistachio, almond, hazelnut, ground nuts, tree nuts, figs, cottonseed	20	4–10 for total 2–5 for B1 0.1 for B1 in baby food
Aflatoxin M_1_	*Metabolite of aflatoxin B1*	2B	Milk and dairy products	0.5	0.05 0.025 baby milk
Fumonisin B_1_, B_2_, B_3_	*Fusarium verticillionides* *Fusarium proliferatum*	2B	Maize, asparagus, corn-based food, white and yellow popcorn, sweet corn	2000–4000	800–1000 200 baby food
Ochratoxin A	*Aspergillus ochraceus* *Penicillium verrucosum* *Aspergillus carbonarius*	2B	Cereals, coffee, cocoa, wine, beer, dried fruits, grapes, pig kidney	Not set	3–10 0.5 baby food
Patulin	*Penicillium expansum*	3	Maize, asparagus, apple, pears, grapes, vegetables, cereals and cheese.	50	25–50 10 baby food
Zearalenone	*Fusarium graminearum* *Fusarium culmorum*	2A	Wheat, corn, barley, oats, sorghum and sesame seeds, hay and corn silage.	Not set	50–100 20 baby food
Deoxynivalenol	*Fusarium graminearum* *Fusarium culmorum*	3	Corn, wheat, oats, barley, rice, grains, beer, animal’s kidney and liver, milk, eggs	1000	750–1250 200 baby food
Nivalenol	*Fusarium graminearum* *Fusarium culmorum*	3	Oats, barley, maize, wheat, bread and fine bakery wares, pasta, cereals	Not set	1.2
T-2 toxin	*Fusarium sporotrichioides*	3	Maize, wheat, corn gluten feed, corn gluten meal, barley, bran.	Not set	0.012–0.043

**Table 3:** Aflatoxin M_1_ quantification by chromatographic methods.

No.	Quantification Method/Detector	Detection Limit/Sample (ppt)	Observations	References
1	Thin Layer Chromatography with Fluorescence Detector (TLC-FD)	100 (non-fat powdered milk) 5 (milk) 100 (beef liver) 12.5 (milk and milk products) 1–15 (cheese)	Clean up: Silica-gel/Reversed C18 column SPE	[76,77,78,79,80]
2	High Performance Liquid Chromatography with Fluorescence Detector (HPLC-FD)	5–35 (raw milk) 0.01–5 (cheese) 600 (white and blue cheese)	Reversed C18 gravity column C18/IAC clean-up	[81,82,83,84,85]
3	High Performance Liquid Chromatography with Mass Spectrometry (HPLC-MS/MS)	50 (milk) 0.59 (whole milk) 0.66 (low fat milk)	SPE-IAC clean-up	[5,58]
4	Liquid Chromatography with Fluorescence Detector (LC-FD)	0.3 (dairy products) 0.8 (human breast milk)	Immunoaffinity column (IAC)	[86]
5	Liquid Chromatography Tandem Mass Spectrometry (LC-MS/MS)	4 (bovine milk) 0.83 (powder milk)	Solid phase extraction (SPE)	[87,88]
6	Ultra High Performance Liquid Chromatography Electrospray Ionisation Tandem Mass Spectrometry (UHPL-ESI/MS)	1 (powder milk) 2 (liquid milk)	SPE	[89]
7	Enzyme-Linked Immunosorbent Assay/High Performance Liquid Chromatography-fluorescence Detector (ELISA/HPLC-FD)	0–13.58 (ELISA in human breast milk) 13.58 (HPLC) >50 (ELISA) 2 (HPLC in milk) 4–31/50 (buffalo and cow milk)	IAC	[52,64,90]
8	Enzyme-Linked Immunosorbent Assay/High Performance Liquid Chromatography-Liquid Chromatography Tandem Mass Spectrometry (ELISA/HPLC-LC-MS)	1.3–6.22 (ELISA) 62.9 (LC-MS in raw and UHT milk)	IAC	[91]
9	Enzyme-Linked Immunosorbent Assay (ELISA)	70.6–770.97 (cheese)	AFM_1_-HRP	[92,93]
10	Electro chemiluminescent-immunoassay	0.3 (milk)	antibody-labeled cadmium telluride quantum dots (CdTe QDs)	
11	Time-resolved fluoro-immunoassay (TRFIA)	0.188 (milk)	AFM_1_-BSA conjugate, anti-AFM_1_ Ab, and Eu-labeled goat anti-rabbit Ab	[94]
12	Sequential injection immunoassay test (SIIA)	200 (milk)		[95]
13	Electrochemical sensing with bilayer lipid membranes (ECS-BLMs)	761 (skimmed milk)	Electrochemical detection	[96]
14	ELISA-SPE (screen-printed electrodes)	25 (milk)	Electrochemical detection	[97]
15	Flow-injection immunoassay	11 (raw milk)	Amperometric detection	[98]
16	Direct chemiluminescent enzyme immunoassay	1 (milk)	Sensitivity improved by using 3-(10′-phenothiazinyl)-propane-1-sulfonate and 4-morpholinopyridine	[99]
17	Immunochip	240 (added in drinking water)	indirect competitive immunoassay	[100]

## References

[1] Sweeney M.J., Dobson A.D.W. (1998). Mycotoxin production by aspergillus, fusarium and penicillium species. Int. J. Food Microbiol..

[2] Marin S., Ramos A.J., Cano-Sancho G., Sanchis V. (2013). Mycotoxins: Occurrence, toxicology, and exposure assessment. Food Chem. Toxicol..

[3] Bennett J.W., Klich M. (2003). Mycotoxins. Clin. Microbiol. Rev..

[4] Mahmoud E.A. (2014). Clinicopathological studies on the effect of fusarium mycotoxin on hematological and biochemical parameters in broiler chickens. Glob. Vet..

[5] Chen Z.Y., Rajasekaran K., Brown R.L., Sayler R.J., Bhatnagar D. (2015). Discovery and confirmation of genes/proteins associated with maize aflatoxin resistance. World Mycotoxin J..

[6] Steyn P.S. (1995). Mycotoxins, general view, chemistry and structure. Toxicol. Lett..

[7] Pitt J.I. (2000). Toxigenic fungi: Which are important?. Med. Mycol..

[8] Bosco F., Mollea C. (2012). Mycotoxins in Food.

[9] Milicevic D.R., Skrinjar M., Baltic T. (2010). Real and perceived risks for mycotoxin contamination in foods and feeds: Challenges for food safety control. Toxins.

[10] Zain M.E. (2011). Impact of mycotoxins on humans and animals. J. Saudi Chem. Soc..

[11] Rodricks J.V. (1977). Mycotoxins in Human and Animal Health.

[12] Stoloff L. (1983). Aflatoxin as a cause of primary liver-cell cancer in the united states: A probability study. Nutr. Cancer.

[13] Ueno Y. (1983). Trichothecenes: Chemical, Biological, and Toxicological Aspects.

[14] Niessen L. (2007). Pcr-based diagnosis and quantification of mycotoxin producing fungi. Int. J. Food Microbiol..

[15] International Agency for Research on Cancer (IARC) (2002). IARC Monographs on the Evaluation of Carcinogenic Risks to Humans.

[16] Mazzoni E., Scandolara A., Giorni P., Pietri A., Battilani P. (2011). Field control of fusarium ear rot, ostrinia nubilalis (hübner), and fumonisins in maize kernels. Pest Manag. Sci..

[17] Wogan G.N. (1992). Aflatoxins as risk-factors for hepatocellular-carcinoma in humans. Cancer Res..

[18] Murphy P.A., Hendrich S., Landgren C., Bryant C.M. (2006). Food mycotoxins: An update. J. Food Sci..

[19] Pereira V.L., Fernandes J.O., Cunha S.C. (2014). Mycotoxins in cereals and related foodstuffs: A review on occurrence and recent methods of analysis. Trends Food Sci. Technol..

[20] Food and Agriculture Organization (FAO) Mycotoxins Food Safety and Quality. http://www.fao.org/food/food-safety-quality/a-zindex/mycotoxins/en/.

[21] European Commission (2001). Commission Regulation (ec) No. 466/2001 Setting Maximum Levels for Certain Contaminants in Foodstuffs.

[22] European Commission (2006). Commission Regulation (EC) No. 1881/2006 Setting Maximum Levels for Certain Contaminants in Foodstuffs.

[23] European Commission (2007). European Commission (EC) No. 1126/2007 Setting Maximum Levels for Certain Contaminants in Foodstuffs as Regards Fusarium Toxins in Maize and Maize Products.

[24] European Commission (2010). Commission Regulation (EU) No 165/2010 of 26 February 2010 Amending Regulation (EC) No 1881/2006 Setting Maximum Levels for Certain Contaminants in Foodstuffs as Regards Aflatoxins.

[25] Alshannaq A., Yu J.-H. (2017). Occurrence, toxicity, and analysis of major mycotoxins in food. Int. J. Environ. Res. Public Health.

[26] Food and Drug Administration (US-FDA) (2011). Mycotoxins.

[27] Bankole S.A., Adebanjo A. (2003). Mycotoxins in food in west africa: Current situation and possibilities of controlling it. Afr. J. Biotechnol..

[28] Gowda N.K.S., Swamy H.V.L.N., Mahajan P., Razzaghi-Abyaneh M. (2013). Recent advances for control, counteraction and amelioration of potential aflatoxins in animal feeds. Aflatoxins—Recent Advances and Future Prospects.

[29] Nguefack J., Leth V., Zollo P.H.A., Mathur S.B. (2004). Evaluation of five essential oils from aromatic plants of cameroon for controlling food spoilage and mycotoxin producing fungi. Int. J. Food Microbiol..

[30] Reddy K.R.N., Nurdijati S.B., Salleh B. (2010). An overview of plant-derived products on control of mycotoxigenic fungi and mycotoxins. Asian J. Plant Sci..

[31] Thembo K.M., Vismer H.F., Nyazema N.Z., Gelderblom W.C.A., Katerere D.R. (2010). Antifungal activity of four weedy plant extracts against selected mycotoxigenic fungi. J. Appl. Microbiol..

[32] Bianchini A., Bullerman L.B. (2009). Biological control of molds and mycotoxins in foods. Mycotoxin Prevention and Control in Agriculture.

[33] Ember L. (1984). Charges of toxic arms use by iraq escalate. Chem. Eng. News.

[34] Vidal J.C., Bonel L., Ezquerra A., Hernández S., Bertolín J.R., Cubel C., Castillo J.R. (2013). Electrochemical affinity biosensors for detection of mycotoxins: A review. Biosens. Bioelectron..

[35] Paterson R.R.M. (2006). Fungi and fungal toxins as weapons. Mycol. Res..

[36] Franz D.R. (1996). Defense Against Toxin Weapons. Med. Asp. Chem. Biol. Warf..

[37] Mejri Omrani N., Hayat A., Korri-Youssoufi H., Marty J.L., Nikolelis D.P., Nikoleli G.-P. (2016). Electrochemical biosensors for food security: Mycotoxins detection. Biosensors for Security and Bioterrorism Applications.

[38] Wilson D.M., Mubatanhema W., Jurjevic Z., DeVries J.W., Trucksess M.W., Jackson L.S. (2002). Biology and ecology of mycotoxigenic aspergillus species as related to economic and health concerns. Mycotoxins and Food Safety.

[39] Shephard G.S. (2009). Aflatoxin analysis at the beginning of the twenty-first century. Anal. Bioanal. Chem..

[40] Prandini A., Tansini G., Sigolo S., Filippi L., Laporta M., Piva G. (2009). On the occurrence of aflatoxin M_1_ in milk and dairy products. Food Chem. Toxicol..

[41] Campagnollo F.B., Ganev K.C., Khaneghah A.M., Portela J.B., Cruz A.G., Granato D., Corassin C.H., Oliveira C.A.F., Sant’Ana A.S. (2016). The occurrence and effect of unit operations for dairy products processing on the fate of aflatoxin M_1_: A review. Food Control.

[42] Santini A., Raiola A., Ferrantelli V., Giangrosso G., Macaluso A., Bognanno M., Galvano F., Ritieni A. (2013). Aflatoxin m-1 in raw, uht milk and dairy products in sicily (Italy). Food Addit. Contam. Part B.

[43] Bartoszek A. (2005). Genotoxic food components. Carcinogenic and Anticarcinogenic Food Components.

[44] Troxel C.M., Reddy A.P., O’Neal P.E., Hendricks J.D., Bailey G.S. (1997). In vivo aflatoxin B_1_ metabolism and hepatic DNA adduction in zebrafish (danio rerio). Toxicol. Appl. Pharmacol..

[45] Ketney O., Ovidiu T., Tifrea A. (2014). Structural diversity and biochemical and microbiological characteristics of aflatoxins. Acta Universitatis Cibiniensis. Series E: Food Technology.

[46] Peers F.G., Linsell C.A. (1973). Dietary aflatoxins and liver cancer—A population based study in kenya. Br. J. Cancer.

[47] Vanrensburg S.J., Cookmozaffari P., Vanschalkwyk D.J., Vanderwatt J.J., Vincent T.J., Purchase I.F. (1985). Hepatocellular-carcinoma and dietary aflatoxin in mozambique and transkei. Br. J. Cancer.

[48] European Food Safety Authority (EFSA) (2007). Opinion of the Scientific Panel on Contaminants in the Food Chain on a Request from the Commission Related to the Potential Increase of Consumer Health Risk by a Possibleincrease of the Existing Maximum Levels for Aflatoxins in Almonds, Hazelnuts and Pistachios and Derived.

[49] Jalili M., Scotter M. (2015). A review of aflatoxin M_1_ in liquid milk. Iran. J. Health Saf. Environ..

[50] El-Tras W.F., El-Kady N.N., Tayel A.A. (2011). Infants exposure to aflatoxin M_1_ as a novel foodborne zoonosis. Food Chem. Toxicol..

[51] Gürbay A., Sabuncuoğlu S.A., Girgin G., Şahin G., Yiğit Ş., Yurdakök M., Tekinalp G. (2010). Aflatoxin M_1_ levels in breast milk samples from ankara, turkey. Toxicol. Lett..

[52] De Roma A., Rossini C., Ritieni A., Gallo P., Esposito M. (2017). A survey on the aflatoxin M_1_ occurrence and seasonal variation in buffalo and cow milk from southern italy. Food Control.

[53] Neal G.E., Eaton D.L., Judah D.J., Verma A. (1998). Metabolism and toxicity of aflatoxins M_1_ and B_1_ in human-derived in vitro systems. Toxicol. Appl. Pharmacol..

[54] Lee N.A., Wang S., Allan R.D., Kennedy I.R. (2004). A rapid aflatoxin B_1_ ELISA: Development and validation with reduced matrix effects for peanuts, corn, pistachio, and soybeans. J. Agric. Food Chem..

[55] Cichna-Markl M. (2011). New strategies in sample clean-up for mycotoxin analysis. World Mycotoxin J..

[56] Kim E.K., Shon D.H., Ryu D., Park J.W., Hwang H.J., Kim Y.B. (2000). Occurrence of aflatoxin M_1_ in korean dairy products determined by ELISA and HPLC. Food Addit. Contam..

[57] Kamkar A. (2006). A study on the occurrence of aflatoxin M_1_ in iranian feta cheese. Food Control.

[58] Wang H., Zhou X.J., Liu Y.Q., Yang H.M., Guo Q.L. (2010). Determination of aflatoxin M_1_ in milk by triple quadrupole liquid chromatography-tandem mass spectrometry. Food Addit. Contam..

[59] Chiavaro E., Cacchioli C., Berni E., Spotti E. (2005). Immunoaffinity clean-up and direct fluorescence measurement of aflatoxins B_1_ and M_1_ in pig liver: Comparison with high-performance liquid chromatography determination. Food Addit. Contam..

[60] Rodríguez Velasco M.L., Calonge Delso M.M., Ordónez Escudero D. (2003). ELISA and HPLC determination of the occurrence of aflatoxin M_1_ in raw cow’s milk. Food Addit. Contam..

[61] Rosi P., Borsari A., Lasi G., Lodi S., Galanti A., Fava A., Girotti S., Ferri E. (2007). Aflatoxin M_1_ in milk: Reliability of the immunoenzymatic assay. Int. Dairy J..

[62] Mwanza M., Abdel-Hadi A., Ali A.M., Egbuta M. (2015). Evaluation of analytical assays efficiency to detect aflatoxin M_1_ in milk from selected areas in egypt and south africa. J. Dairy Sci..

[63] Bognanno M., La Fauci L., Ritieni A., Tafuri A., De Lorenzo A., Micari P., Di Renzo L., Ciappellano S., Sarullo V., Galvano F. (2006). Survey of the occurrence of aflatoxin M_1_ in ovine milk by HPLC and its confirmation by MS. Mol. Nutr. Food Res..

[64] Bellio A., Bianchi D., Gramaglia M., Loria A., Nucera D., Gallina S., Gili M., Decastelli L. (2016). Aflatoxin M_1_ in cow’s milk: Method validation for milk sampled in northern italy. Toxins.

[65] Cavaliere C., Foglia P., Pastorini E., Samperi R., Laganà A. (2006). Liquid chromatography/tandem mass spectrometric confirmatory method for determining aflatoxin M_1_ in cow milk. J. Chromatogr. A.

[66] De Iongh H.R.V., de Vogel P., Wogan G.H. (1964). The occurrence and detection of aflatoxin in food. Proceedings of the Symposium on Mycotoxins in Foodstuffs.

[67] Betina V. (1993). Chapter 7 thin-layer chromatography of mycotoxins. J. Chromatogr. Libr..

[68] Gulyás H. (1985). Determination of aflatoxins B_1_, B_2_, G_1_, G_2_ and M_1_ by high pressure thin layer chromatography. J. Chromatogr..

[69] Stroka J., Anklam E. (2002). New strategies for the screening and determination of aflatoxins and the detection of aflatoxin-producing moulds in food and feed. Trends Anal. Chem..

[70] Lin L., Zhang J., Wang P., Wang Y., Chen J. (1998). Thin-layer chromatography of mycotoxins and comparison with other chromatographic methods. J. Chromatogr. A.

[71] Li P., Zhang Q., Zhang W. (2009). Immunoassays for aflatoxins. Trends Anal. Chem..

[72] Shuib N.S., Makahleh A., Salhimi S.M., Saad B. (2017). Determination of aflatoxin M_1_ in milk and dairy products using high performance liquid chromatography-fluorescence with post column photochemical derivatization. J. Chromatogr. A.

[73] Joshua H. (1993). Determination of aflatoxins by reversed-phase high-performance liquid chromatography with post-column in-line photochemical derivatization and fluorescence detection. J. Chromatogr. A.

[74] Chiavaro E., Dall’Asta C., Galaverna G., Biancardi A., Gambarelli E., Dossena A., Marchelli R. (2001). New reversed-phase liquid chromatographic method to detect aflatoxins in food and feed with cyclodextrins as fluorescence enhancers added to the eluent. J. Chromatogr. A.

[75] Kos J., Hajnal E.J., Jajic I., Krstovic S., Mastilovic J., Saric B., Jovanov P. (2016). Comparison of ELISA, HPLC-FLD and HPLC-MS/MS methods for determination of aflatoxin M_1_ in natural contaminated milk samples. Acta Chim. Slov..

[76] Fukayama M., Winterlin W., Hsieh D.P. (1980). Rapid method for analysis of aflatoxin M_1_ in dairy products. J. Assoc. Off. Anal. Chem..

[77] Gauch R., Leuenberger U., Baumgartner E. (1979). Rapid and simple determination of aflatoxin M_1_ in milk in the low parts per 1012 range. J. Chromatogr. A.

[78] Bakirci I. (2001). A study on the occurrence of aflatoxin M_1_ in milk and milk products produced in van province of turkey. Food Control.

[79] Bijl J., van Peteghem C. (1985). Rapid extraction and sample clean-up for the fluorescence densitometric determination of aflatoxin M_1_ in milk and mil powder. Anal. Chim. Acta.

[80] Kamkar A. (2005). A study on the occurrence of aflatoxin M_1_ in raw milk produced in sarab city of iran. Food Control.

[81] Boudra H., Barnouin J., Dragacci S., Morgavi D.P. (2007). Aflatoxin M_1_ and ochratoxin a in raw bulk milk from french dairy herds. J. Dairy Sci..

[82] Decastelli L., Lai J., Gramaglia M., Monaco A., Nachtmann C., Oldano F., Ruffier M., Sezian A., Bandirola C. (2007). Aflatoxins occurrence in milk and feed in northern italy during 2004–2005. Food Control.

[83] Kokkonen M., Jestoi M., Rizzo A. (2005). Determination of selected mycotoxins in mould cheeses with liquid chromatography coupled to tandem with mass spectrometry. Food Addit. Contam..

[84] Manetta A.C., Di Giuseppe L., Giammarco M., Fusaro I., Simonella A., Gramenzi A., Formigoni A. (2005). High-performance liquid chromatography with post-column derivatisation and fluorescence detection for sensitive determination of aflatoxin m-1 in milk and cheese. J. Chromatogr. A.

[85] Mao J., Lei S., Liu Y., Xiao D., Fu C., Zhong L., Ouyang H. (2015). Quantification of aflatoxin M_1_ in raw milk by a core-shell column on a conventional HPLC with large volume injection and step gradient elution. Food Control.

[86] Iha M.H., Barbosa C.B., Okada I.A., Trucksess M.W. (2011). Occurrence of aflatoxin M_1_ in dairy products in brazil. Food Control.

[87] Sørensen L.K., Elbæk T.H. (2005). Determination of mycotoxins in bovine milk by liquid chromatography tandem mass spectrometry. J. Chromatogr. B.

[88] Chew Y.L., Xing J., Lim L.G.S., Zhan Z. A High Sensitivity LC/MS/MS Method with QuEChERS Sample Pre-treatment for Analysis of Aflatoxins in Milk Powder Samples. https://www.ssi.shimadzu.com/about/literature/asms2016/wednesday/wP-230.pdf.

[89] Huang L.C., Zheng N., Zheng B.Q., Wen F., Cheng J.B., Han R.W., Xu X.M., Li S.L., Wang J.Q. (2014). Simultaneous determination of aflatoxin M_1_, ochratoxin a, zearalenone and α-zearalenol in milk by UHPLC–MS/MS. Food Chem..

[90] Afshar P., Shokrzadeh M., Kalhori S., Babaee Z., Saravi S.S.S. (2013). Occurrence of ochratoxin a and aflatoxin M_1_ in human breast milk in sari, iran. Food Control.

[91] Bilandzic N., Tankovic S., Jelusic V., Varenina I., Kolanovic B.S., Luburic D.B., Cvetnic Z. (2016). Aflatoxin M_1_ in raw and uht cow milk collected in bosnia and herzegovina and croatia. Food Control.

[92] Kav K., Col R., Tekinsen K.K. (2011). Detection of aflatoxin M_1_ levels by ELISA in white-brined urfa cheese consumed in turkey. Food Control.

[93] Gan N., Zhou J., Xiong P., Hu F., Cao Y., Li T., Jiang Q. (2013). An ultrasensitive electrochemiluminescent immunoassay for aflatoxin M_1_ in milk, based on extraction by magnetic graphene and detection by antibody-labeled cdte quantumn dots-carbon nanotubes nanocomposite. Toxins.

[94] Guo M., Zhou B., Huang Z., Zhao C., Zhang J., Huang B. (2017). A new method for determination of alfatoxin M_1_ in milk by ultrasensitive time-resolved fluoroimmunoassay. Food Anal. Methods.

[95] Garden S.R., Strachan N.J.C. (2001). Novel colorimetric immunoassay for the detection of aflatoxin b-1. Anal. Chim. Acta.

[96] Andreou V.G., Nikolelis D.P., Tarus B. (1997). Electrochemical investigation of transduction of interactions of aflatoxin M_1_ with bilayer lipid membranes (blms). Anal. Chim. Acta.

[97] Micheli L., Grecco R., Badea M., Moscone D., Palleschi G. (2005). An electrochemical immunosensor for aflatoxin M_1_ determination in milk using screen-printed electrodes. Biosens. Bioelectron..

[98] Badea M., Micheli L., Messia M.C., Candigliota T., Marconi E., Mottram T., Velasco-Garcia M., Moscone D., Palleschi G. (2004). Aflatoxin M_1_ determination in raw milk using a flow-injection immunoassay system. Anal. Chim. Acta.

[99] Vdovenko M.M., Lu C.C., Yu F.Y., Sakharov I.Y. (2014). Development of ultrasensitive direct chemiluminescent enzyme immunoassay for determination of aflatoxin M_1_ in milk. Food Chem..

[100] Wang Y., Liu N., Ning B., Liu M., Lv Z., Sun Z., Peng Y., Chen C., Li J., Gao Z. (2012). Simultaneous and rapid detection of six different mycotoxins using an immunochip. Biosens. Bioelectron..

[101] Wacoo A.P., Wendiro D., Vuzi P.C., Hawumba J.F. (2014). Methods for detection of aflatoxins in agricultural food crops. J. Appl. Chem..

[102] Sargent A., Sadik O.A. (1999). Monitoring antibody—Antigen reactions at conducting polymer-based immunosensors using impedance spectroscopy. Electrochim. Acta.

[103] Wang X., Niessner R., Tang D., Knopp D. (2016). Nanoparticle-based immunosensors and immunoassays for aflatoxins. Anal. Chim. Acta.

[104] Dinckaya E., Kinik O., Sezginturk M.K., Altug C., Akkoca A. (2011). Development of an impedimetric aflatoxin M_1_ biosensor based on a DNA probe and gold nanoparticles. Biosens. Bioelectron..

[105] Dimitrieska-Stojkovic E., Stojanovska-Dimzoska B., Ilievska G., Uzunov R., Stojkovic G., Hajrulai-Musliu Z., Jankuloski D. (2016). Assessment of aflatoxin contamination in raw milk and feed in macedonia during 2013. Food Control.

[106] Kunter İ., Hürer N., Gülcan H.O., Öztürk B., Doğan İ., Şahin G. (2017). Assessment of aflatoxin M_1_ and heavy metal levels in mothers breast milk in famagusta, cyprus. Biol. Trace Elem. Res..

[107] Liu B.H., Chu K.C., Yu F.Y. (2016). Novel monoclonal antibody-based sensitive enzyme-linked immunosorbent assay and rapid immunochromatographic strip for detecting aflatoxin M_1_ in milk. Food Control.

[108] Paek S.H., Lee S.H., Cho J.H., Kim Y.S. (2000). Development of rapid one-step immunochromatographic assay. Methods.

[109] Parker C.O., Tothill I.E. (2009). Development of an electrochemical immunosensor for aflatoxin M_1_ in milk with focus on matrix interference. Biosens. Bioelectron..

[110] Radoi A., Targa M., Prieto-Simon B., Marty J.L. (2008). Enzyme-linked immunosorbent assay (ELISA) based on superparamagnetic nanoparticles for aflatoxin M_1_ detection. Talanta.

[111] Wang J.-J., Liu B.-H., Hsu Y.-T., Yu F.-Y. (2011). Sensitive competitive direct enzyme-linked immunosorbent assay and gold nanoparticle immunochromatographic strip for detecting aflatoxin M_1_ in milk. Food Control.

[112] Lamberti L.M.A.I., Torres-Pacheco I. (2011). Biosensors for aflatoxins detection. Aflatoxins—Detection, Measurement and Control.

[113] Maragos C. (2009). Fluorescence polarization immunoassay of mycotoxins: A review. Toxins.

[114] Jie G., Li L., Chen C., Xuan J., Zhu J.-J. (2009). Enhanced electrochemiluminescence of cdse quantum dots composited with cnts and pdda for sensitive immunoassay. Biosens. Bioelectron..

[115] Liu X.A., Zhang Y.Y., Lei J.P., Xue Y.D., Cheng L.X., Ju H.X. (2010). Quantum dots based electrochemiluminescent immunosensor by coupling enzymatic amplification with self-produced coreactant from oxygen reduction. Anal. Chem..

[116] McAllister M.J., Li J.L., Adamson D.H., Schniepp H.C., Abdala A.A., Liu J., Herrera-Alonso M., Milius D.L., Car R., Prud’homme R.K. (2007). Single sheet functionalized graphene by oxidation and thermal expansion of graphite. Chem. Mater..

[117] Ricci F., Volpe G., Micheli L., Palleschi G. (2007). A review on novel developments and applications of immunosensors in food analysis. Anal. Chim. Acta.

[118] Ricci F., Adornetto G., Palleschi G. (2012). A review of experimental aspects of electrochemical immunosensors. Electrochim. Acta.

[119] Karczmarczyk A., Baeumner A.J., Feller K.-H. (2017). Rapid and sensitive inhibition-based assay for the electrochemical detection of ochratoxin a and aflatoxin M_1_ in red wine and milk. Electrochim. Acta.

[120] Paniel N., Radoi A., Marty J.L. (2010). Development of an electrochemical biosensor for the detection of aflatoxin m-1 in milk. Sensors.

[121] Bacher G., Pal S., Kanungo L., Bhand S. (2012). A label-free silver wire based impedimetric immunosensor for detection of aflatoxin M_1_ in milk. Sens. Actuator B Chem..

[122] Banerjee P., Kintzios S., Prabhakarpandian B. (2013). Biotoxin detection using cell-based sensors. Toxins.

[123] Valimaa A.L., Kivisto A.T., Leskinen P.I., Karp M.T. (2010). A novel biosensor for the detection of zearalenone family mycotoxins in milk. J. Microbiol. Methods.

[124] Larou E., Yiakoumettis I., Kaltsas G., Petropoulos A., Skandamis P., Kintzios S. (2013). High throughput cellular biosensor for the ultra-sensitive, ultra-rapid detection of aflatoxin M_1_. Food Control.

[125] Ellington A.D., Szostak J.W. (1990). In vitro selection of rna molecules that bind specific ligands. Nature.

[126] Tuerk C., Gold L. (1990). Systematic evolution of ligands by exponential enrichment: Rna ligands to bacteriophage t4 DNA polymerase. Science.

[127] Jayasena S.D. (1999). Aptamers: An emerging class of molecules that rival antibodies in diagnostics. Clin. Chem..

[128] Nguyen B.H., Tran L.D., Do Q.P., Nguyen H.L., Tran N.H., Nguyen P.X. (2013). Label-free detection of aflatoxin M_1_ with electrochemical Fe_3_O_4_/polyaniline-based aptasensor. Mater. Sci. Eng. C.

[129] Malhotra S., Pandey A.K., Rajput Y.S., Sharma R. (2014). Selection of aptamers for aflatoxin M_1_ and their characterization. J. Mol. Recognit..

[130] Istamboulie G., Paniel N., Zara L., Granados L.R., Barthelmebs L., Noguer T. (2016). Development of an impedimetric aptasensor for the determination of aflatoxin M_1_ in milk. Talanta.

[131] Szalontai H., Kiss A., Adanyi N. (2014). Determination of aflatoxin M_1_ in milk samples by an owls-based immunosensor. Acta Aliment..

[132] Lou X., Zhu A., Wang H., Wu J., Zhou L., Long F. (2016). Direct and ultrasensitive optofluidic-based immunosensing assay of aflatoxin M_1_ in dairy products using organic solvent extraction. Anal. Chim. Acta.

[133] Zhang Z., Li P., Hu X., Zhang Q., Ding X., Zhang W. (2012). Microarray technology for major chemical contaminants analysis in food: Current status and prospects. Sensors.

